# A case of palisaded neutrophilic granulomatous dermatitis with subsequent development of chronic myelomonocytic leukemia

**DOI:** 10.1002/ccr3.2072

**Published:** 2019-02-20

**Authors:** Aikaterini Kyriakou, Aikaterini Patsatsi, Vassilios Papadopoulos, Anna Kioumi, Ioannis Efstratiou, Elizabeth Lazaridou

**Affiliations:** ^1^ 2nd Department of Dermatology and Venereology, General Hospital “Papageorgiou”, Medical School Aristotle University of Thessaloniki Thessaloniki Greece; ^2^ Hematology Department General Hospital “Papageorgiou” Thessaloniki Greece; ^3^ Pathology Department General Hospital “Papageorgiou” Thessaloniki Greece

**Keywords:** connective tissue, lymphoproliferative, rheumatoid arthritis, sarcoidosis

## Abstract

Palisaded neutrophilic granulomatous dermatitis is a cutaneous marker of a systemic disease. Clinicians’ goal should be directed toward determining an underlying condition. Even if the initial investigation is inconclusive, it may be necessary that some tests are repeated, since a serious underlying disease could be revealed in the course of time.

## 1 INTRODUCTION

Palisaded neutrophilic granulomatous dermatitis (PNGD) is an infrequent histopathological diagnosis usually associated with an underlying systemic disease,^1^ such as connective tissue disease, rheumatoid arthritis, sarcoidosis, lymphoproliferative disorder, vasculitis, infection, and inflammatory bowel disease.^2^, ^3^ Infrequently, PNGD may be either idiopathic, with no identified cause, or drug‐induced.^4^, ^5^, ^6^ PNGD may precede or occur concomitantly with the underlying disease.^1^, ^7^ Its clinical presentation varies; however, PNGD usually presents as erythematous to violaceous papules or plaques with central umbilication or necrosis,^7^ mostly distributed symmetrically on the extensor surfaces of the upper extremities, head, or neck.^8^ A case of a patient with skin lesions that were histopathologically compatible with PNGD is presented, with subsequent development of chronic myelomonocytic leukemia (CMML).

## 2 CASE HISTORY/EXAMINATION

**Figure 1:**
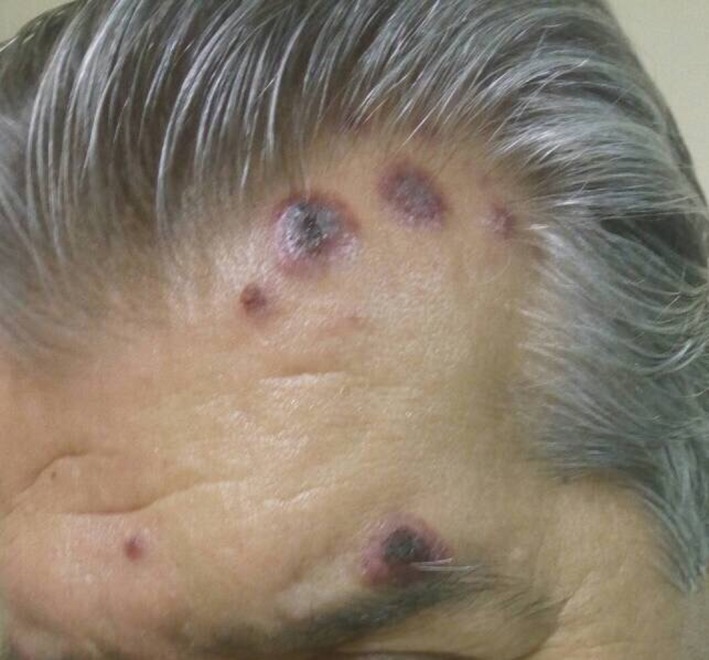
Violaceous, indurated plaques located on the head

A 59‐year‐old male was admitted to our Department with a 3‐month history of fatigue, fevers, and unintentional weight loss over this period. He also reported a 2‐week history of violaceous, mildly tender, indurated plaques located on the extensor surfaces of the upper extremities, and head (Figure 1).

## 3 DIFFERENTIAL DIAGNOSIS, INVESTIGATIONS, AND TREATMENT

**Figure 2:**
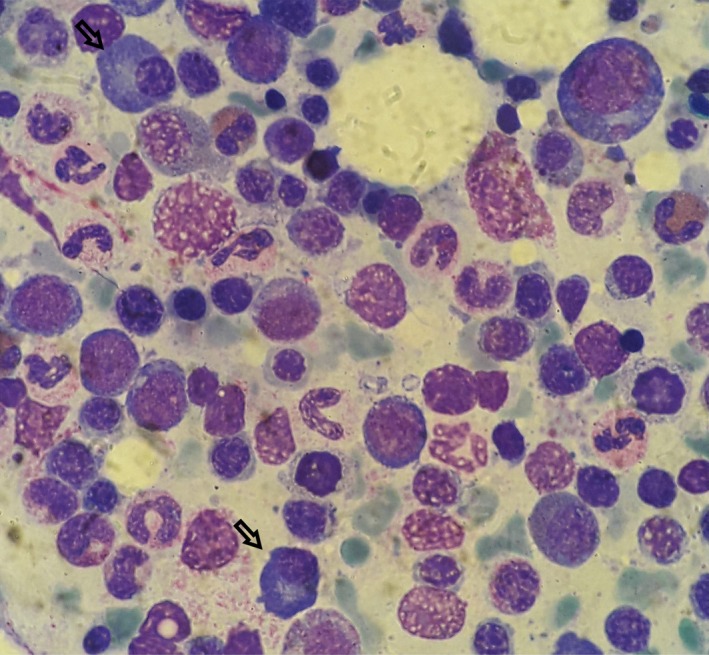
Initial bone marrow smear showing sparse plasma cells (arrows) and reactive changes—May‐Grunwald‐Giemsa (MGG) stain x 100

**Figure 3:**
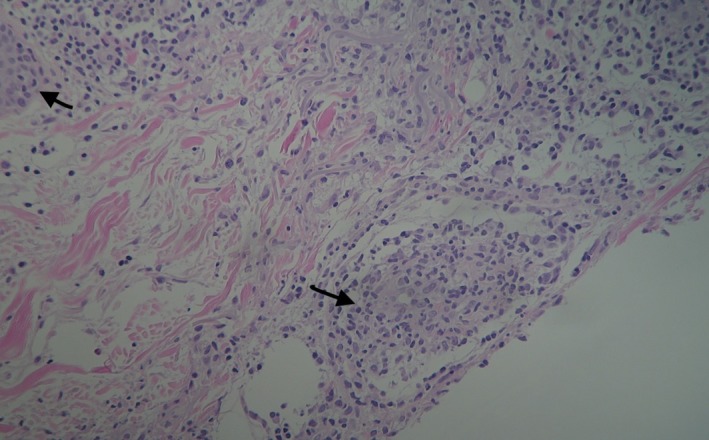
Formation of granulomas—H&E X 10

**Figure 4:**
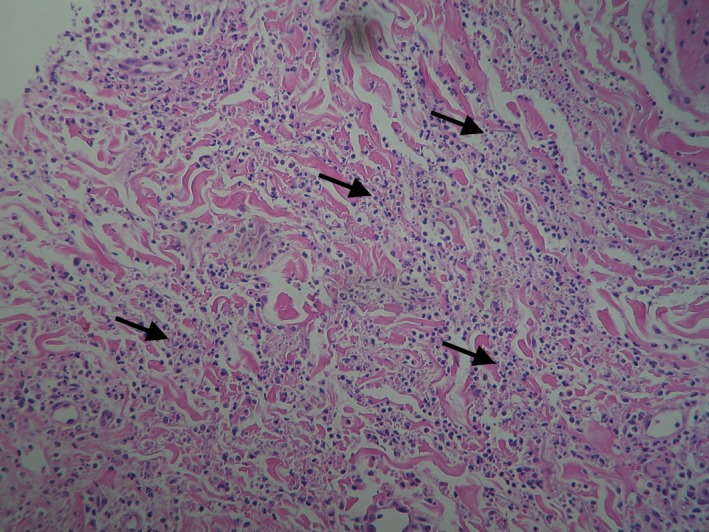
Neutrophilic infiltration and nuclear debris—H&E X 40

The patient had no significant medical history and received no medications regularly. Over the last 3 months, plenty of tests had been performed to investigate the fever of unknown origin. A full blood count had revealed normocytic anemia with leukocytosis and monocytosis, as well as immature granulocytes in the peripheral blood smear (WBC: 14.4 × 10^9^/L, neutrophils/lymphocytes/monocytes: 57/23/18%, absolute counts: 8.18, 3.37, 2.59 × 10^9^/L respectively, hemoglobin: 102 G/L, platelets: 234 × 10^9^/L). He had also had elevated inflammatory markers (CRP: 12 mg/dL). Liver function tests had been normal, except for mildly elevated lactate dehydrogenase (LDH: 267 IU/L). The patient had been tested negative for a number of autoimmune and infective diseases. At initial presentation, bone marrow biopsy had revealed a small percentage (15%) of nonclonal plasma cells, suggestive of an extra‐medullary disease (Figure 2). During the referral to our Department, a skin biopsy was performed and revealed lymphocytes and eosinophils, palisading granulomas, and neutrophilic debris (Figures 3 and 4), which was compatible with the diagnosis of PNGD. Subsequently, the patient was followed closely with repeated blood smears.

## 4 OUTCOME AND FOLLOW‐UP

**Figure 5:**
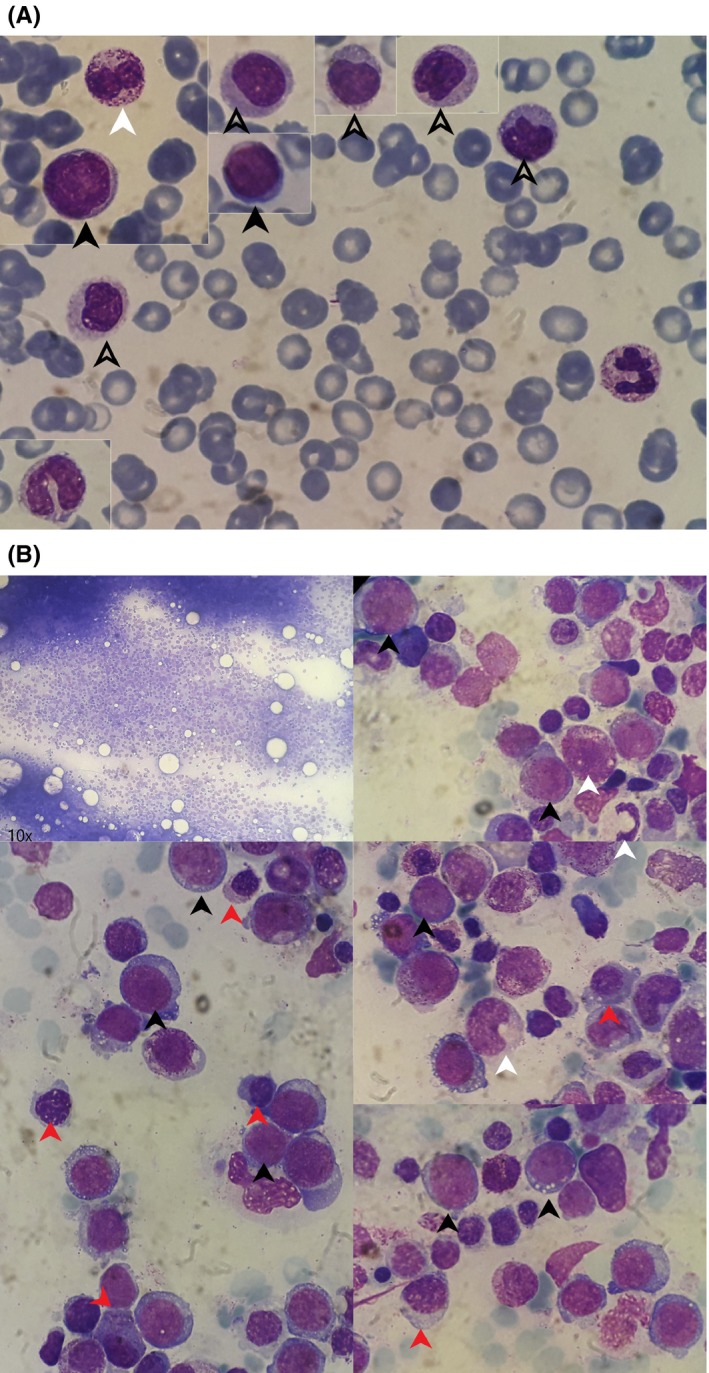
A, Peripheral blood on follow‐up suggestive of CMML. B, Bone marrow smear on follow‐up suggestive of CMML. Arrowheads: abnormal monocytes, black arrowheads: blasts, white arrowhead: dysplastic neutrophil, red arrowheads: dysplastic erythroblasts. (MGG x100). B, upper left image (x10) depicts increased cellularity

During the follow‐up, the anemia gradually deteriorated and transfusions of red blood cells were required, while thrombocytopenia was developed. After 5 months from the skin biopsy, the complete blood counts were as follows: WBC: 12.7 × 10^9^/L, neutrophils: 4.45 × 10^9^/L, lymphocytes 1 × 10^9^/L, monocytes: 4.7 × 10^9^/L, Hb: 75 G/L, PLT: 60 × 10^9^/L (Figure 5A). Subsequently, another bone marrow biopsy was conducted, which revealed greatly increased cellularity, presence of 14% myeloblasts and 6% monocytes, and morphological dysplasia of erythroid and megakaryocytic lineage (Figure 5B). After exclusion of other myeloproliferative neoplasms (JAK2 ‐ V617F, BCR/ABL negative), and according to WHO‐2018 criteria, the diagnosis of CMML‐2 was reached. Τhe cytogenetic analysis of bone marrow showed complex karyotype and peripheral blood flow cytometry further supported the diagnosis. Unfortunately, the patient passed away a few days after the diagnosis of his hematologic condition, due to cardiac arrest. No treatment for his condition had ever been initiated.

## 5 DISCUSSION

Pathogenesis of PNGD remains poorly understood. Direct immunofluorescence studies have suggested immune complex deposition.^9^ Lately, it has been suggested that the granulomas may represent a nonspecific immunological response possibly related to the underlying disease.^10^

Palisaded neutrophilic granulomatous dermatitis proceeds through different histologic stages^9^; thus, clincopathologic correlation is compulsory.^8^, ^11^A single biopsy may not initially reveal the combination of findings indicative of PNGD.^11^ Early lesions show diffuse neutrophils with or without leukocytoclastic vasculitis and degenerated collagen; fully developed lesions present palisaded granulomas surrounding leukocytoclastic debris, and altered collagen.^1^, ^12^ In our case, the patient showed fully developed lesions characterized histologically by the presence of neutrophilic infiltration and nuclear debris, as well as granulomas.

Palisaded neutrophilic granulomatous dermatitis is benign disease and its management is based on the control of the underlying disease.^13^ However, plenty therapeutic options have been reported such as systemic corticosteroids, colchicine, cyclosporine, cyclophosphamide, hydroxychloroquine, and dapsone.^2^ In our case, no action was taken, since the patient passed away a few days after the diagnosis of his hematologic condition, due to cardiac arrest.

The association between PNGD and hematological malignancies has been reported constantly,^8^, ^14^, ^15^ Therefore, it is strongly recommended to differentiate it from leukemic infiltrates.^14^ Infiltration of the skin by leukemic cells is quite rare in CMML and may predict a rapid aggressive course and a shift to a blast transformation of the disease.^16^, ^17^ Cutaneous lesions may present as erythematous rashes, plaques, nodules, or pigmented nodules without any typical clinical features and with heterogeneous histopathologic features.^16^, ^17^

The concurrence of PNGD and CMML has been reported recently by Federmann et al, who presented three patients with disseminated lesions histopathologically consistent with PNGD and persistent monocytosis.^14^ An important aspect of our case is that the cutaneous lesions were one of the first clinical complaints in our patient contributing significantly to the diagnosis of his hematological disorder.^14^

The identification of PNGD is of great importance, since it is a cutaneous marker of systemic disease.^10^ Clinicians’ goal should be directed toward determining an underlying condition.^4^, ^8^ Even if the initial investigation is inconclusive, it may be necessary that some tests are repeated, since a serious underlying disease could be revealed in the course of time.

## CONFLICT OF INTEREST

None declared.

## AUTHOR CONTRIBUTION

AK, VP: collected clinical data and wrote the manuscript. AP, AK, VP, IE, EL: contributed to patient's evaluation and follow‐up. AP, EL: reviewed the manuscript. All authors read and approved the final version of the manuscript.
